# The effect of gut microbiota elimination in *Drosophila melanogaster*: A how‐to guide for host–microbiota studies

**DOI:** 10.1002/ece3.3991

**Published:** 2018-03-26

**Authors:** Chloe Heys, Anne Lizé, Frances Blow, Lewis White, Alistair Darby, Zenobia J. Lewis

**Affiliations:** ^1^ School of Life Sciences/Institute of Integrative Biology University of Liverpool Liverpool UK; ^2^ UMR 6553 ECOBIO University of Rennes Rennes France

**Keywords:** *Drosophila*, life‐history traits, microbiota, physiology

## Abstract

In recent years, there has been a surge in interest in the effects of the microbiota on the host. Increasingly, we are coming to understand the importance of the gut microbiota in modulating host physiology, ecology, behavior, and evolution. One method utilized to evaluate the effect of the microbiota is to suppress or eliminate it, and compare the effect on the host with that of untreated individuals. In this study, we evaluate some of these commonly used methods in the model organism, *Drosophila melanogaster*. We test the efficacy of a low‐dose streptomycin diet, egg dechorionation, and an axenic or sterile diet, in the removal of gut bacteria within this species in a fully factorial design. We further determine potential side effects of these methods on host physiology by performing a series of standard physiological assays. Our results showed that individuals from all treatments took significantly longer to develop, and weighed less, compared to normal flies. Males and females that had undergone egg dechorionation weighed significantly less than streptomycin reared individuals. Similarly, axenic female flies, but not males, were much less active when analyzed in a locomotion assay. All methods decreased the egg to adult survival, with egg dechorionation inducing significantly higher mortality. We conclude that low‐dose streptomycin added to the dietary media is more effective at removing the gut bacteria than egg dechorionation and has somewhat less detrimental effects to host physiology. More importantly, this method is the most practical and reliable for use in behavioral research. Our study raises the important issue that the efficacy of and impacts on the host of these methods require investigation in a case‐by‐case manner, rather than assuming homogeneity across species and laboratories.

## 1 INTRODUCTION

In the past few years, there has been an explosion of interest in the gut microbiota and the myriad ways in which it affects host processes from modulating immune responses (Round & Mazmanian, 2009) to mate selection (Lizé, McKay, & Lewis, 2014). To date, using a Web of Science search, there have been 4,617 articles published on the gut microbiota, across diverse species (search terms: gut microbio* under TITLE). Of that number, 3,281 (71%) of these were published in the last 4 years. However, there is little consensus regarding the most effective method for eliminating the gut microbiota, despite its importance for our understanding of the effects the gut microbiota may have on the host.

Drosophilid species, particularly *Drosophila melanogaster,* have become an important model for examining how changes to, or differences in, the gut microbiota affect the host, for example, by regulating intestinal regeneration (Buchon, Broderick, & Lemaitre, 2013), or through driving mating preferences (Sharon et al., 2010). For such studies to be considered reliable, effective methods of altering the gut microbiota must be utilized in concordance with a given study system.

In *Drosophila*, there are two particularly common methods of altering gut bacterial communities: supplementing dietary media with antibiotics or creating sterile or axenic flies using egg dechorionation. The protective outer layer of the egg, the chorion, is coated with highly diverse bacteria transmitted largely from fecal deposits from the mother during oviposition (Wong, Ng, & Douglas, 2011). Emerging larvae then ingest the chorion and the bacteria coating it, forming the basis of their microbial community (Bakula, 1967). Removal of this embryonic chorion using bleach creates axenic, or microbe‐free, adults. Supplementing the dietary media with antibiotics is a considerably simpler method. Here, a broad‐spectrum antibiotic such as streptomycin or tetracycline is added to the diet; some studies use a combination of antibiotics in order to remove the microbiota (Sharon, Segal, Zilber‐Rosenberg, & Rosenberg, 2011; Sharon et al., 2010).

Both the use of antibiotics and dechorionation of the egg are widely applied and widely criticized. Therefore, evaluating the efficacy of current methods and how they impact the study organism is vital for the investigation of host–microbiota relationships. Some recent publications have favored the use of antibiotics (Sharon et al., 2010, 2011). Yet while broad‐spectrum antibiotics are active against a wide range of bacterial species, they also act on host enzymes and mitochondrial proteins by inhibiting synthesis, and/or nucleic acid metabolism and repair (Brodersen et al., 2000). In pseudoscorpions, this has been shown to reduce sperm viability, and the effect can be passed down generations (Zeh, Bonilla, Adrian, Mesfin, & Zeh, 2012). The repeated use of broad‐spectrum antibiotics also has severe consequences in other organisms. For example, in humans, long‐term antibiotic use is thought to correlate, directly or indirectly, with diseases such as type 2 diabetes and early‐life obesity (Blaser & Falkow, 2009). Egg dechorionation in egg‐laying animals is thought to be a less hazardous method of eliminating gut bacteria. However, studies comparing this with antibiotic treatment have only ever used harsher antibiotics such as chlortetracycline or rifampicin and in high concentrations (Ridley, Wong, Westmiller, & Douglas, 2012). The impacts on the host of tetracycline use have been fairly well documented (e.g. O'Shea & Singh, 2015; Zeh et al., 2012), yet to date, little attention has focused on low‐dose streptomycin.

**Figure 1:**
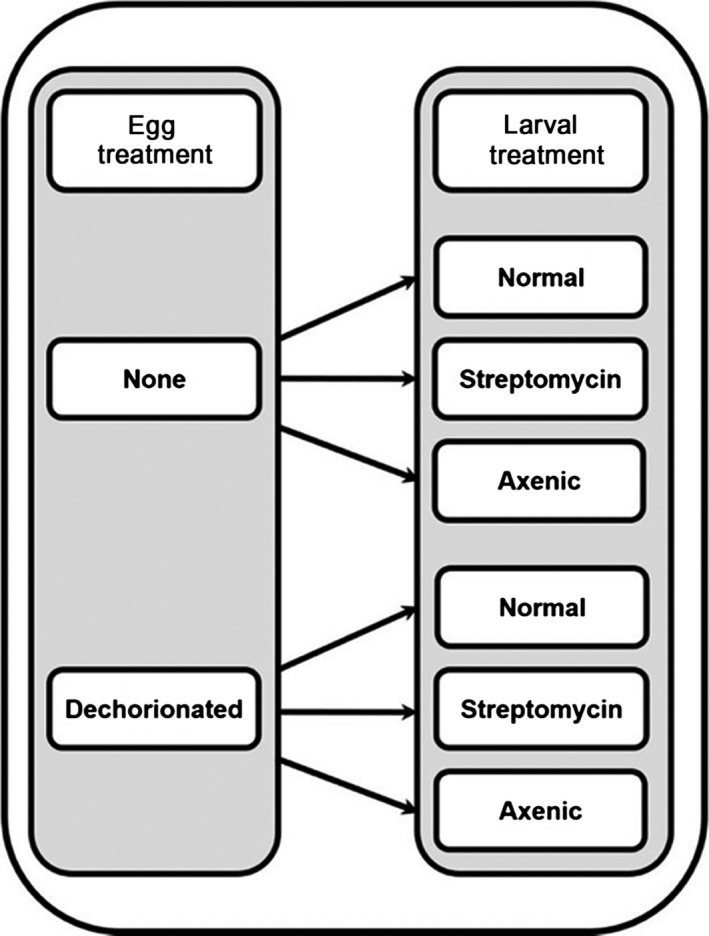
Schematic representation of our 2 × 3 factorial design of egg and larval treatments. Physiological assays were conducted on flies from each treatment type

In this study, we analyzed the efficacy and the physiological effects on the flies, of the most common methods used to eliminate the resident host gut microbiota in *D. melanogaster*. We compared flies reared via a range of methods, in a factorial design: those reared on streptomycin, egg‐dechorionated individuals, and flies reared on an axenic diet (Figure 1). Parallel to bacterial analyses determining the effectiveness of the techniques in eliminating the gut microbiota, we conducted a series of standard physiological assays in order to test the effect of each treatment on the overall health and fitness of the fly host. We measured development time from egg to adulthood (Tantway & El‐Helw, 1970), adult weight (Partridge & Fowler, 1993), egg to adult survival, and how adults responded to stress. In the natural environment, the ability of *D. melanogaster* to develop more quickly on the limited food source of rotting fruit is beneficial to survival, as it ensures an individual can achieve pupation before the food source is exhausted (Nunney, 1996). This pressure is also increased if multiple females lay eggs on the same fruit. Thus, measuring development time is a fundamental assay of an individual's physiological fitness. Similarly, size directly correlates with mating success in *Drosophila*, with larger males being more successful (Partridge & Farquhar, 1983). Starvation assays measure how long a fly can survive when deprived of nutrition (Service, Hutchinson, Mackinley, & Rose, 1985), while locomotion assays such as the rapid iterative negative geotaxis (RING) assay (Gargano, Martin, Bhandari, & Grotewiel, 2005) measure the innate escape response, where individuals ascend the walls of a container after being knocked to the bottom. From these results, we suggest addition of antibiotics to the diet is the most effective method for eliminating the gut microbiota in our *Drosophila* system, with the least deleterious effects for the host. We note that this method is both more practical and reliable when conducting behavioral experiments, as, when using axenic individuals, there is a high likelihood of introducing external bacteria through the very nature of manipulating the study organisms. Our results demonstrate the importance of considering the potential impacts of each method with respect to the host organism studied, and the target research area.

## 2 MATERIALS AND METHODS

### 2.1 Fly stocks

Wild‐type, *Wolbachia*‐free *D. melanogaster* stocks were isolated from an outbred population collected in Lyon. Flies were reared at 25°C under a 12:12‐hour light:dark cycle. Recently mated females were placed into vials containing 25 ml standard yeast–cornmeal diet (for 1 L of water: 85 g of sugar, 60 g of corn, 20 g of yeast, 10 g of agar, and 25 ml of nipagin) and left to lay eggs for 24 hr. The following day, the females were removed and eggs were collected using a fine paintbrush. The eggs and their successive developing larvae were then assigned to one of the six treatments (Figure 1). Henceforth, we abbreviate our treatments as outlined in Table 1.

**Table 1:** Treatment abbreviations used throughout and sample sizes for each

Treatments	Abbreviations	Egg to adult survival/	Weight		Starvation		Response to stress (rapid iterative negative geotaxis assay)	
		Development time	Males	Females	Males	Females	Males	Females
Eggs reared normally Larvae reared normally	N‐Norm	1,100/438	68	87	97	104	35	25
Eggs reared normally Larvae reared on streptomycin food	N‐Strep	1,100/353	62	62	90	95	25	25
Eggs reared normally Larvae reared on axenic food	N‐Ax	1,400/188	53	59	60	51	25	25
Eggs dechorionated Larvae reared normally	D‐Norm	1,050/152	80	67	54	52	30	25
Eggs dechorionated Larvae reared on streptomycin food	D‐Strep	1,100/127	68	56	53	54	25	25
Eggs dechorionated Larvae reared on axenic food	D‐Ax	1,450/82	50	50	51	53	25	25

Once eggs had been harvested and a treatment assigned (e.g. dechorionated or not), they were placed into vials at a standard density of fifty per vial. Eggs that were not subjected to the dechorionation were still physically manipulated in the same way, but without the chemical treatment. Thus, we controlled for any potential effects of physically manipulating the eggs, across all treatments. Eggs were then left to hatch, and the emergent larvae left to develop. At eclosion, newly emerged adults were isolated using an aspirator and separated according to sex. Males and females were stored separately in groups of 10 in vials containing 25 ml of the diet on which they were reared as larvae.

### 2.2 Experimental treatments

#### 2.2.1 Normal diet

Eggs assigned to a normal diet treatment were transferred into vials containing 25 ml standard yeast–cornmeal diet at 25°C and left to develop.

#### 2.2.2 Diet containing streptomycin

Once harvested from the stock vials, eggs were then transferred into vials containing 25 ml standard yeast–cornmeal diet that had been supplemented with streptomycin at a concentration of 400 μg/ml, as is common in the literature (Lizé et al., 2014; Sharon et al., 2010). Upon cooling, 4 ml of a solution composed of 10 g of streptomycin in 100 ml of ethanol was added per liter of food. Food was then dispensed into vials with 25 ml in each.

#### 2.2.3 Axenic diet

An axenic diet was produced by autoclaving vials of standard yeast–cornmeal diet, without the addition of nipagin, for 20 min at 120°C. Nipagin was added once the media had cooled to 65°C. Any manipulation of the axenic diet was conducted under a laminar flow cabinet to ensure sterility. Twenty‐five milliliter of the media was then dispensed into sterile vials.

#### 2.2.4 Egg dechorionation

Eggs were gently harvested using a sterile paintbrush and placed onto a piece of fine cloth mesh. They were then placed into a strainer and washed with sterile, deionized water once. They were then immersed in a 10% sodium hypochlorite solution for 5 min (Ridley et al., 2012). The eggs were washed three more times with sterile, deionized water and then carefully removed using a sterile paintbrush and placed onto the desired food treatment. All work was conducted under a laminar flow cabinet to ensure sterility. Eggs from all treatments were subjected to the physical manipulation utilized during the egg dechorionation treatment, but without the addition of bleach, in order to control for any deleterious effects of the action.

### 2.3 Physiological assays

#### 2.3.1 Development time

Once treated, eggs were placed into the development time assay and the number of days for these eggs to emerge as newly eclosed adults was counted. Vials were checked at three time points within each day—9 a.m., 12 p.m., and 5 p.m.—and the cumulative number of adults emerged from each time point was scored. Emergent adult flies from each time point were removed from the vial and placed into a fresh vial of their corresponding diet treatment.

#### 2.3.2 Egg to adult survival

Each vial was set up to contain fifty eggs so that the number of flies that reach adulthood could be counted. Vials were checked at three time points within each day—9 a.m., 12 p.m., and 5 p.m.—and the cumulative number of alive, newly eclosed adult flies was counted. Emergent flies were then removed from the vial and placed into a fresh vial of their corresponding diet treatment. This was repeated daily until there were no live larvae or pupae left in the vial. The mortality rate was then calculated from the number of flies that had reached adulthood compared to the number of eggs set up.

#### 2.3.3 Adult weight

Vials were checked daily at three time points—9 a.m., 12 p.m., and 5 p.m.—and any newly emerged, virgin adults were isolated and separated according to sex. They were placed into vials at a standard density of 10 per vial and left for 2 hr to allow their wings to dry out and inflate. Flies reared in the egg dechorionation treatments and the axenic larval treatments were always manipulated within the laminar flow cabinet in order to prevent contamination. Two hours later, vials were placed into the freezer at −18°C and left overnight. The following morning, individuals were collected from the freezer using a Kahn balance and their weight was recorded (in mg) to four decimal places. Male and female measurements for each treatment were recorded and analyzed separately.

#### 2.3.4 Starvation resistance

Newly emerged, virgin adults were isolated and separated according to sex. Flies reared in the egg dechorionation treatments, and the axenic larval treatments were always manipulated within the laminar flow cabinet in order to prevent contamination. Flies were placed into vials at a standard density of 10 per vial and left to mature for 2 days. After this time, they were transferred to a fresh vial containing 10 ml of non‐nutritional agar in order to prevent desiccation. Fresh agar was used to prevent microorganismal growth—no bacterial and fungal growth was observed during the course of the experiment. Flies were left in these vials to acclimatize for 24 hr, and then, the starvation assay was started. The time to starvation death was measured by monitoring the flies every 8 hr—at 8 a.m., 4 p.m., and 12 a.m.. Here, the number of dead flies was counted and the starvation assay continued until there were no more living flies. This assay was conducted at 25°C. Male and female measurements for each treatment were recorded and analyzed separately.

#### 2.3.5 Locomotion—RING (rapid iterative negative geotaxis)

Newly emerged, virgin adults were isolated and separated according to sex. Flies reared in the egg dechorionation treatments, and the axenic larval treatments were always manipulated within a laminar flow cabinet in order to prevent contamination. Flies were placed into vials at a standard density of 10 per vial and left to mature for 2 days. After this time, flies were placed into fresh vials containing 10 ml of the diet type on which they were reared. Five vials were then placed into an apparatus similar to that described by Gargano et al. (2005) and Nichols, Becnel, and Pandey (2012), and flies were left to acclimatize for 30 min. After this time, the apparatus was rapped sharply on the work surface three times in rapid succession in order to initiate the negative geotaxis response. After a 3‐s rest, a photograph was taken of the vials, recording the flies’ position within the vial and thus their negative geotaxis response to the stimulus. After a 1‐min rest, the procedure was repeated. This procedure was repeated five times in total for each set of flies, resulting in five digital images for each vial. This assay was performed at 25°C. Male and female measurements for each treatment were recorded and analyzed separately.

Digital images were later analyzed manually by measuring the distance each fly had travelled following the tapping stimulus. All 10 flies in each vial were measured across the five digital images generated.

An average distance travelled value was then created for each vial and statistical analysis performed.

### 2.4 Bacterial analysis

In order to quantify the bacterial load within flies reared on each treatment, and therefore the efficacy of each treatment, we cultured the bacteria present in both the whole gut and the whole fly. Newly emerged, virgin adults were isolated and separated according to sex. Flies reared in the egg dechorionation treatments and the axenic larval treatments were always manipulated within a laminar flow cabinet in order to prevent contamination. Flies were placed into vials at a standard density of 10 per vial and left to mature for 2 days.

#### 2.4.1 Gut bacterial analysis

Following maturation, adults were isolated using gas anesthesia and surface‐sterilized in 70% ethanol, rinsed in distilled water, and air‐dried. The head was then removed. Three guts were dissected into each Eppendorf containing 500 μl of sterile PBS (phosphate‐buffered saline solution). An equal number of males and females were used in order to ensure there were no sex‐specific differences in the bacterial content. Gut tissue was homogenized with a sterile plastic pestle. One hundred microliter of gut homogenate was pipetted onto MRS (de Man, Rogosa and Sharpe) agar and spread‐plated using a sterile glass loop. Plates were left to air‐dry aseptically, before being closed and sealed with parafilm. Plates were incubated at 25°C for 72 hr, and bacterial load was quantified by performing colony‐forming unit (CFU) counts.

#### 2.4.2 Whole‐fly bacterial analysis

Following maturation, flies were isolated using gas anesthesia and placed into a sterile Eppendorf containing 500 μl sterile PBS. Three flies were added into each Eppendorf. An equal number of males and females were used in order to ensure there were no sex‐specific differences in the bacterial content. The whole‐fly solute was then homogenized using a sterile, plastic pestle. One hundred microliter of the whole‐fly solute was pipetted into the center of a petri dish containing MRS media and spread across the plate using a sterile glass loop. The plate was left to dry close to the flame before being closed and sealed using parafilm. Plates were incubated at 25°C for 72 hr and then checked for bacterial growth. Bacterial load was quantified by performing CFU counts.

Single colonies were isolated using a sterile 1 μl loop and placed into an Eppendorf with 10 μl sterile water. PCR amplification was performed in a 25 μl reaction volume consisting of 10 μl nuclease‐free water, 13 μl Taq green master mix, 0.5 μl of forward primer 27F (5′‐AGAGTTTGATCMTGGCTCAG‐3′) and reverse primer 1492R (5′‐GGTTACCTTGTTACGACTT‐3′), and 1 μl of template DNA. Thermal cycling was performed for 90 s at 95°C as initial denaturation, followed by 35 cycles of 30 s at 95°C for denaturation, 30 s at 55°C as annealing, 90 s at 72°C for extension, and final extension at 72°C for 5 min. One thousand five hundred base pair 16S PCR products were purified with Ampure beads and subjected to Sanger sequencing. The resulting sequences were identified using NCBI BLAST against the nt database (Altschul, Gish, Miller, Myers, & Lipman, 1990).

### 2.5 Statistical analysis

Sample sizes are given in Table 1. All analyses were performed in R (3.1.3) (Ihaka & Gentleman, 1996), and the effects of egg (dechorionation or not) and larval treatments (normal, axenic, and streptomycin) were studied in addition to their interactions. Egg to adult survival, weight, and response to stress (RING assay) were analyzed by fitting a General Linear Model with binomial, Gaussian, and Gaussian distributions, respectively. Weight data were Box–Cox‐transformed to improve normality of the GLM residuals. All GLMs were followed by an ANOVA to test for global effects, and post hoc multiple comparisons between treatments were conducted using Tukey's HSD tests. Following these general GLMs, sexes were studied separately for weight and response to stress (starvation and RING assay).

Cox proportional hazard regressions for survival were used to assess variation in development time and survival under starvation. Survival analysis involves the modeling of time to event data, with death being considered the “event.” Death and development failure of flies was used as the “event” for survival data and development time data, respectively. The *Survdiff* function was used to assess differences between two or more survival curves according to egg and larval treatments. The *coxph* function was used to assess differences between treatments. This allowed treatments to be compared in a pairwise fashion, to ascertain whether all treatments differed or whether any significant differences observed were derived from a single treatment.

## 3 RESULTS

### 3.1 Development time

**Figure 2:**
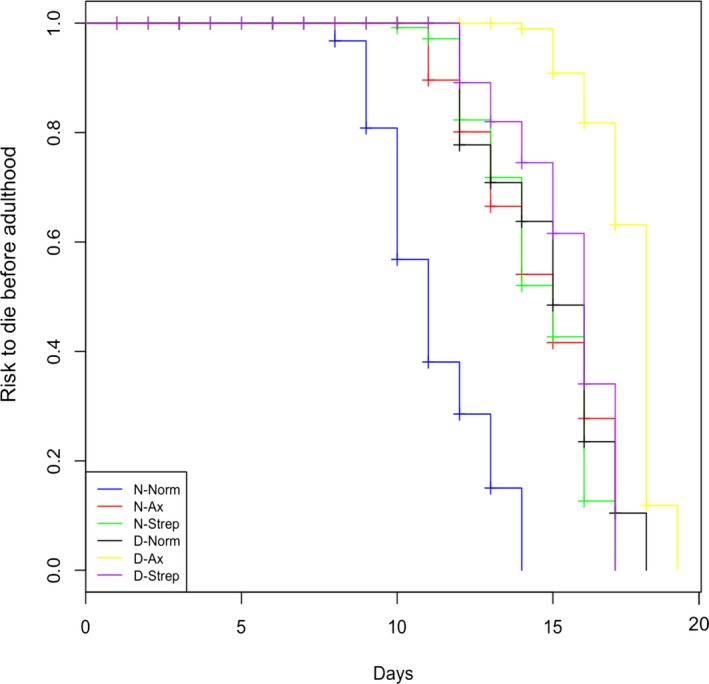
Development time failure measured as the risk to die before adulthood over time in days when eggs were dechorionated (D), or not (N), and when larvae were reared in a conventional diet (Norm), an axenic diet (Ax), or an antibiotic‐supplemented diet (Strep)

Globally, egg dechorionation (*Surdiff*, $\chi_{1}^{1}$ = 473, *p* < .001) and larval treatments (*Surdiff*, $\chi_{2}^{1}$ = 726, *p *<* *.001) altered fly development time (Figure 2). When compared to N‐Norm flies, egg dechorionation (*Coxph*, β ± *SE* = 0.068 ± 0.101, *Z* = −26.305, *p*_z_ < .001) and larval treatments (*Coxph*, Ax, β ± *SE* = 0.091 ± 0.093, *Z* = −25.603, *p*_z_ < .001, Strep, β ± *SE* = 0.089 ± 0.077, *Z* = −31.110, *p *<* *.001) increased development time of flies. Moreover, egg dechorionation and larval treatment effects interacted with each other (*Coxph*, D‐Ax, β ± *SE* = 1.461 ± 0.173, *Z* = 2.187, *p*_z_ = .028, D‐Strep, β ± *SE* = 8.406 ± 0.143, *Z* = 14.875, *p*_z_ < .001). Thus, removing or altering the microbiota increased development time.

### 3.2 Egg to adult survival

**Figure 3:**
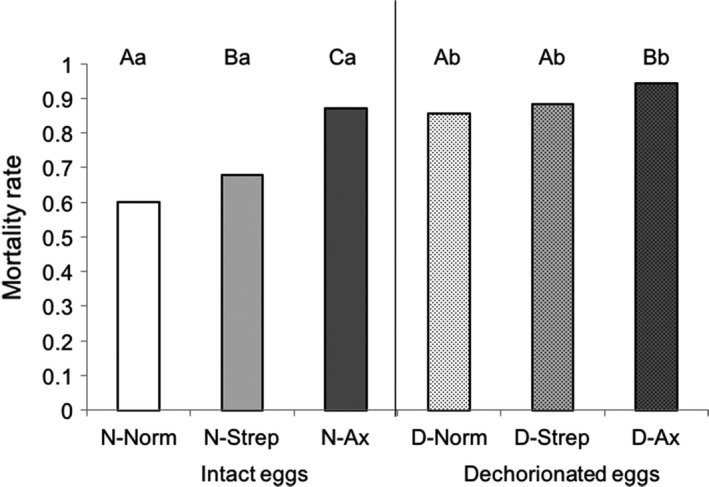
Egg to adult survival measured as mortality rate when eggs were dechorionated (D) or not (N), and when larvae were reared conventionally (Norm), or with the antibiotic streptomycin (Strep), or with axenic media (Ax). Different uppercase letters represent significant differences between larval treatments within egg treatment, while different lowercase letters represent significant differences within larval treatment between egg treatments

Globally, across all treatments, dechorionation (*p *<* *.001) and larval treatments (*p *<* *.001) affected egg to adult survival both as factors and via interaction (*p *=* *.024; Figure 3). More specifically, larval treatments (Ax and Strep) significantly increased mortality during development compared to Norm when eggs were intact (N‐Norm–N‐Ax: *p *<* *.001, N‐Norm–N‐Strep: *p *<* *.001, N‐Ax–N‐Strep: *p *<* *.001). In dechorionated eggs, only the Ax treatment increased mortality during development compared to Norm and Strep (D‐Norm–D‐Ax: *p *<* *.001, D‐Norm–D‐Strep: *p *=* *.434, D‐Ax–D‐Strep: *p *=* *.011). Furthermore, egg dechorionation also increased mortality within larval treatments (N‐Norm–D‐Norm: *p *<* *.001, N‐Strep–D‐Strep: *p *<* *.001, and N‐Ax–D‐Ax: *p *<* *.001).

In this assay, it should be noted that egg to adult survival for nondechorionated eggs and conventionally reared larvae is quite low (mortality rate of 60%) compared to previous studies where egg to adult viability is approximately 100% (Kristensen et al., 2015). However, as nondechorionated eggs were manipulated the same way as dechorionated eggs, but without the chemical agents to remove the chorion, we are confident that the results are comparable.

### 3.3 Weight

**Figure 4:**
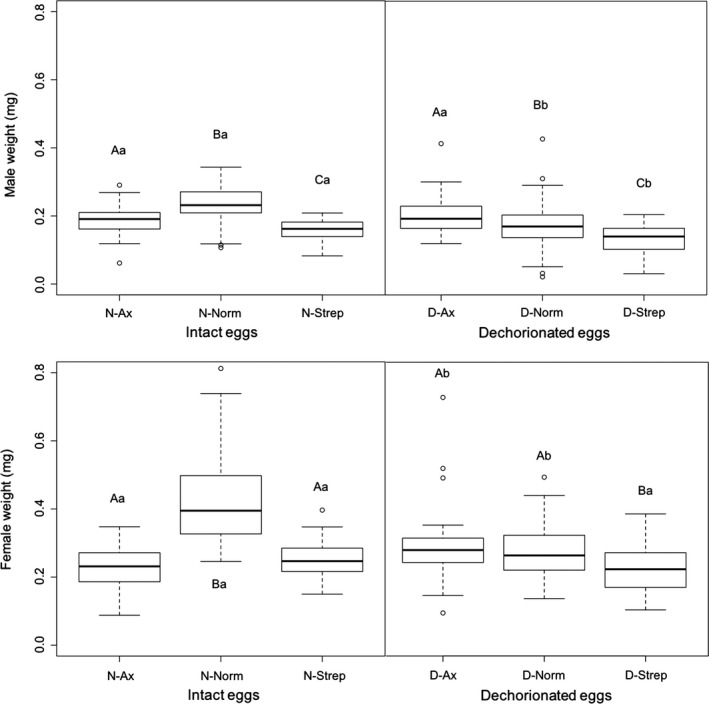
Boxplot of adult male and female weight according to egg treatments (dechorionated [D] or not [N]), and larval treatments (conventionally reared [Norm], axenic medium [Ax], or antibiotic‐supplemented medium [Strep]). Different uppercase letters represent significant differences between larval treatments within egg treatment, while different lowercase letters represent significant differences within larval treatment between egg treatments

Unsurprisingly, adult males were always found to weigh less than adult females across all treatments (*p *<* *.001). When males and females are treated separately, dechorionation (*p *<* *.001) and larval treatments (*p *<* *.001) affected male adult weight both as factors and via interaction (*p *=* *.024; Figure 4). In intact eggs, Ax and Strep larval treatments significantly decreased male adult weight compared to Norm (N‐Norm–N‐Ax: *p *<* *.001, N‐Norm–N‐Strep: *p *<* *.001, N‐Ax–N‐Strep: *p *=* *.011). By contrast, in dechorionated eggs, Ax treatment increased male adult weight when compared to Norm (D‐Ax–D‐Norm: *p *=* *.011) and Strep (D‐Ax–D‐Strep: *p *<* *.001), while Strep decreased male adult weight when compared to Norm (D‐Strep–D‐Norm: *p *<* *.001). Furthermore, egg dechorionation also decreased male adult weight within larval treatments (N‐Norm–D‐Norm: *p *<* *.001, N‐Strep–D‐Strep: *p *=* *.020), except for Ax (N‐Ax–D‐Ax: *p *=* *.928).

In females, dechorionation (*p *<* *.001) and larval treatments (*p *<* *.001) affected female adult weight both as factors and via interaction (*p *<* *.001; Figure 4). In intact eggs, Ax and Strep larval treatments significantly decreased female adult weight compared to Norm (N‐Norm–N‐Ax: *p *<* *.001, N‐Norm–N‐Strep: *p *<* *.001), while Ax had no effect on female adult weight compared to Strep (N‐Ax–N‐Strep: *p *=* *.372). In dechorionated eggs, only the Strep larval treatment significantly decreased female adult weight compared to Norm (D‐Norm–D‐Strep: *p *=* *.019) or Ax (D‐Ax–D‐Strep: *p *=* *.009), while Ax larval treatment had no significant impact on female adult weight (D‐Norm–D‐Ax: *p *=* *.997). Furthermore, egg dechorionation decreased female adult weight within the Norm treatment (N‐Norm–D‐Norm: *p *<* *.001), while increasing it within the Ax treatment (N‐Ax–D‐Ax: *p *=* *.006), but egg dechorionation had no effect within the Strep treatment (N‐Strep–D‐Strep: *p *=* *.448).

### 3.4 Starvation

As expected, males and females did not react the same way to starvation stress, with males dying more quickly than females (*Coxph*, β ± *SE* = 0.424 ± 0.193, *Z* = −4.431, *p*_z_ < .001). Thus, males and females were analyzed separately.

**Figure 5:**
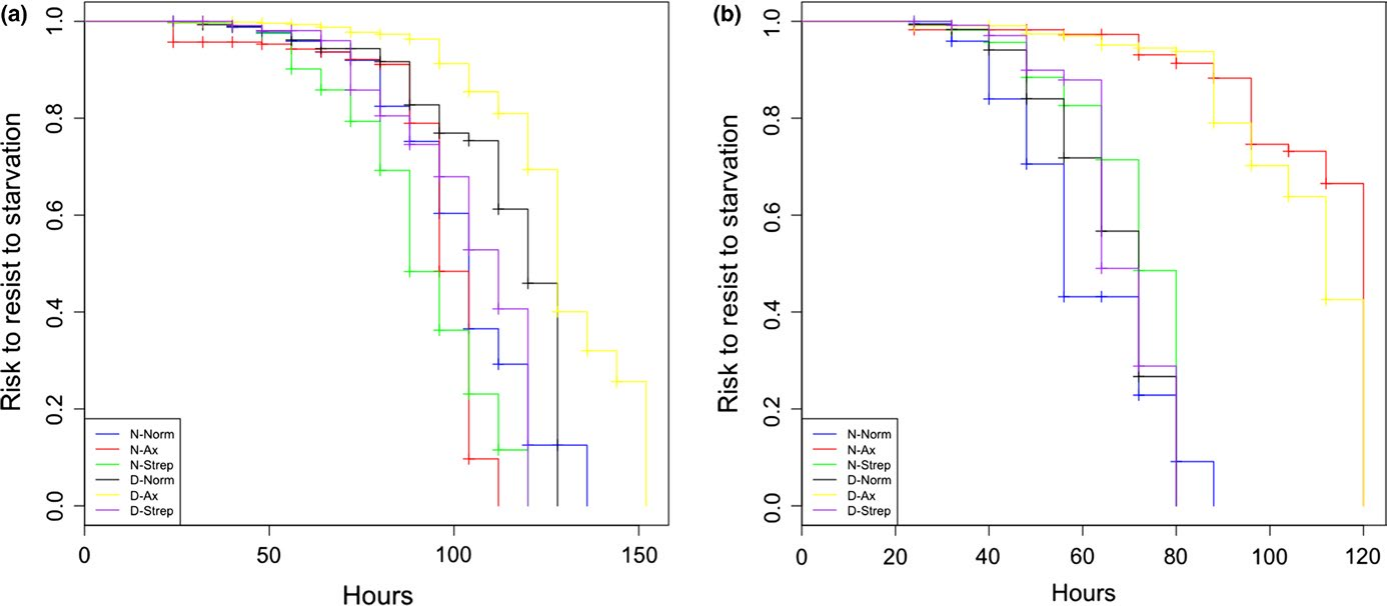
Female (a) and male (b) survival curves representing the risk to resist starvation over time in hours when eggs were dechorionated (D), or not (D), and reared as larvae in a conventional diet (Norm), an axenic diet (Ax), or an antibiotic‐supplemented diet (Strep)

In females, egg dechorionation (*Surdiff,* $\chi_{2}^{1}$ = 117, *p *<* *.001) as well as larval treatments (*Surdiff,* $\chi_{3}^{2}$ = 90.6, *p* < .001) affected female survival (Figure 5a). Egg dechorionation increased female resistance to starvation (*Coxph*, β ± *SE* = 0.508 ± 0.172, *Z* = −3.918, *p*_z_ < .001). Axenic rearing of the larvae had no significant impact on female resistance to starvation when compared to conventionally reared larvae (*Coxph*, β ± *SE* = 1.379 ± 0.172, *Z* = 1.864, *p*_z_ = .062). In contrast, antibiotic rearing of the larvae decreased female resistance to starvation when compared with conventionally reared larvae (*Coxph*, β ± *SE* = 2.092 ± 0.144, *Z* = 5.122, *p*_z_ < .001).

In males, egg dechorionation had no significant impact on male resistance to starvation (*Surdiff,* $\chi_{2}^{1}$ = 1.1, *p *=* *.291; Figure 5b). In contrast, larval treatments affected male resistance to starvation (*Surdiff,* $\chi_{3}^{2}$ = 450, *p *<* *.001), with axenic rearing of the larvae (*Coxph*, β ± *SE* = 2.191 ± 0.257, *Z* = 3.050, *p*_z_ = .002) in addition to antibiotic rearing of the larvae (*Coxph*, β ± *SE* = 2.162 ± 0.245, *Z* = 3.146, *p*_z_ = .001), increasing male resistance to starvation when compared to conventionally reared larvae.

### 3.5 Response to stress (RING assay)

Global effects show that sex (*p *=* *.311) had no significant effect on fly locomotion. However, sex interacted significantly with larval treatments (*p *<* *.001) in determining fly locomotion. Therefore, we treated males and females separately.

**Figure 6:**
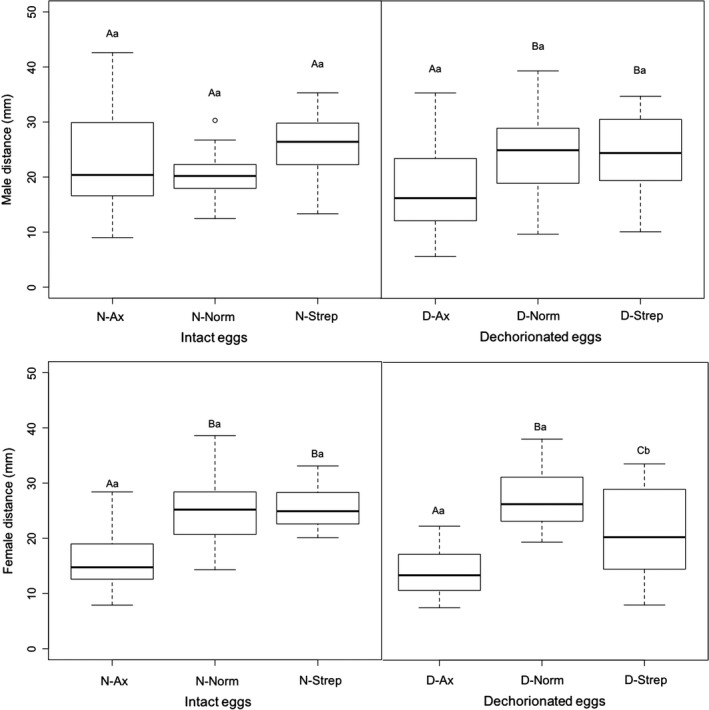
Boxplot of male and female locomotion, measured as distance travelled (RING) according to egg treatments (dechorionated (D) or not (N), and larval treatments (conventionally reared [Norm], axenic medium [Ax], or antibiotic‐supplemented media [Strep]). Different uppercase letters represent significant differences between larval treatments within egg treatment, while different lowercase letters represent significant differences within larval treatment between egg treatments

In males, larval treatments (*p *=* *.001) affected their locomotion as a factor and via an interaction with egg treatments (*p *=* *.001), while egg treatment as a factor had no significant effect on male locomotion (*p *=* *.988; Figure 6). In intact eggs, Ax and Strep larval treatments had no significant effect on male locomotion (N‐Norm–N‐Ax: *p *=* *.913, N‐Norm–N‐Strep: *p *=* *.051, N‐Strep–N‐Ax: *p *=* *.518). By contrast, in dechorionated eggs, Ax significantly reduced male locomotion compared to Norm (D‐Ax–D‐Norm: *p *=* *.001) or Strep (D‐Ax–D‐Strep: *p *=* *.006), while no significant effects on male locomotion was found for Strep when compared to Norm (D‐Norm–D‐Strep: *p *=* *.999). Furthermore, egg dechorionation had no effect on male locomotion within larval treatments (N‐Norm–D‐Norm: *p *=* *.090, N‐Ax–D‐Ax: *p *=* *.153, N‐Strep–D‐Strep: *p *=* *.990).

In females, larval treatments (*p *=* *.001) affected their locomotion as a factor and via an interaction with egg treatments (*p *=* *.004), while egg treatment as a factor had no significant effect on female locomotion (*p *=* *.139; Figure 6). In intact eggs, Ax larval treatment significantly reduced female locomotion when compared to Norm (N‐Norm–N‐Ax: *p *<* *.001) and Strep (N‐Strep–N‐Ax: *p *=* *.001), while Strep had no effect on female locomotion when compared to Norm (N‐Norm–N‐Strep: *p *=* *.999). In dechorionated eggs, both Ax and Strep larval treatments significantly reduced female locomotion compared to Norm (D‐Ax–D‐Norm: *p *<* *.001, D‐Strep–D‐Norm: *p *<* *.001, D‐Ax–D‐Strep: *p *<* *.001). Furthermore, egg dechorionation had no effect within Norm and Ax larval treatments (N‐Norm–D‐Norm: *p *=* *.545, N‐Ax–D‐Ax: *p *=* *.829), while it significantly decreased female locomotion within the Strep larval treatment (N‐Strep–D‐Strep: *p *=* *.032).

### 3.6 Bacterial analysis

In order to assess the efficacy of each treatment in eliminating the gut microbiota, we dissected the midgut of adult *D. melanogaster* and used spread plates on to MRS media to determine the contents. We analyzed the bacterial content of the midgut as this is one of the only larval structures that stays intact during pupation. It is known that a sharp decrease in bacterial density occurs 24 hr after pupation, only increasing again after 48 hr (Storelli et al., 2011), but the midgut is contained and develops within a transient pupal epithelium (Takashima, Younossi‐Hartenstein, Ortiz, & Hartenstein, 2011). As the midgut remains unchanged during pupation while almost all other structures are histolyzed, the midgut is an accurate representative of the gut bacterial content and diversity within an adult *Drosophila*. We also analyzed the bacterial content of the whole fly in a similar manner in order to determine whether our treatments affected the whole host–microbiota. We used colony‐forming unit (CFU) counts to measure the bacterial load of flies from each treatment in triplicate by taking the average, which is a standard measure of estimating bacterial load (Nadkani, Martin, Jacques, & Hunter, 2002).

We discovered bacterial colony growth on all plates from all treatments, except those from flies reared on the streptomycin diet alone. In the case of the latter, there were zero colonies present on all spread plates containing the dissected midgut. For D‐Strep flies, only one of three replicate midgut plates contained any colony growth (Table 2), with the other two replicates containing zero colonies. This is likely an anomaly due to potential contamination of the media during spread plating, or transfer of bacteria from other parts of the fly during midgut dissection.

**Table 2:** Number of bacterial colonies

Treatments	Origin of bacteria	Average number of bacterial cells per gut in each replicate
N‐Norm	Gut	3.1 × 10^1^
	Gut	5.9 × 10^1^
	Gut	6.2 × 10^1^
	Whole fly	4.5 × 10^2^
	Whole fly	3.8 × 10^2^
	Whole fly	6.3 × 10^2^
N‐Strep	Gut	0
	Gut	0
	Gut	0
	Whole fly	2.1 × 10^1^
	Whole fly	2.8 × 10^1^
	Whole fly	1.0 × 10^2^
N‐Ax	Gut	1.8 × 10^2^
	Gut	3.3 × 10^2^
	Gut	2.0 × 10^2^
	Whole fly	5.5 × 10^2^
	Whole fly	5.7 × 10^2^
	Whole fly	4.4 × 10^2^
D‐Norm	Gut	5.5 × 10^1^
	Gut	2.8 × 10^1^
	Gut	8.3 × 10^1^
	Whole fly	1.4 × 10^2^
	Whole fly	7.3 × 10^1^
	Whole fly	6.4 × 10^2^
D‐Strep	Gut	0
	Gut	0
	Gut	0.4 × 10^1^
	Whole fly	0
	Whole fly	4.5 × 10^1^
	Whole fly	2.1 × 10^1^
D‐Ax	Gut	0.04 × 10^1^
	Gut	1.0 × 10^1^
	Gut	7.6 × 10^1^
	Whole fly	6.2 × 10^2^
	Whole fly	5.3 × 10^2^
	Whole fly	4.5 × 10^2^

The results for the midgut contrast with the results of the whole‐fly spread plates, in which colony growth occurs on all replicates for both the N‐Strep and the D‐Strep flies (Table 2), although it can be noted that these results are considerably lower compared to all other treatments. Considerably more colonies were found for the whole‐fly spread plates for each treatment in comparison with the midgut contents. The highest number of colonies was found on the normal treatment, which is to be expected (Table 2). Yet similar numbers of bacterial colonies were found for the whole‐fly plates from the axenic and the combined egg dechorionation and axenic treatment.

## 4 DISCUSSION

Effectively eliminating the resident gut microbiota is essential to the study of host–microbiota interactions, through which we can gain a greater understanding of a species’ fundamental ecology. From the array of physiological assays conducted, it is clear that manipulating the microbiota has a profound effect on the overall health of the host. This is particularly true for development time and adult weight; individuals from all treatments took significantly longer to develop, and weighed less, compared to normal flies. This is hardly surprising considering the gut microbiota is known to affect a wealth of host developmental and physiological processes (Sommer & Backhed, 2013). In *D. melanogaster*, a symbiotic relationship exists between the fly and its gut microbe, *Acetobacter pomorum* (Shin et al., 2011). Acetic acid produced by the alcohol dehydrogenase of *A. pomorum* initiates insulin signaling and thereby tunes the homeostatic signaling of the fly, controlling a variety of factors included developmental rate and body size.

In terms of mortality rate of individuals, considerably fewer flies survived to adulthood when reared on axenic and streptomycin diets compared with normal flies. Sterilization of the diet by rendering it axenic had the most profound effect on egg to adult survival. Removal of the egg chorion also increased mortality rate in all larval treatments (Norm, Strep, and Ax). Dechorionation involved the use of bleach and alcohol to remove the chorion, which acts as a barrier to the environment, and protects against dehydration in insects such as coleopterans (Biémont, Chauvin, & Hamon, 1981) and dipterans (Klowden, 2013). Thus, dechorionation in itself (i.e. the absence of the barrier) might explain the higher mortality rate observed. Sterilization or antibiotic supplementation of the diet kills all or part of the bacteria present in the diet that are ingested by the flies. These bacteria could be used as a food source by the flies and/or help the flies in digesting complex carbohydrates present in the diet, as shown by previous studies (Storelli et al., 2011; Wong et al., 2015). Some of the treated flies could thus have died due to poor nutrition and/or inability to develop through their life cycle. Our findings contrast to previous studies that found that dechorionation had no effect on survivorship from egg to adulthood (Ridley, Wong, & Douglas, 2013). The stark differences in these results highlight the importance for individual laboratories to evaluate the impacts of the methods employed to remove or alter the microbiota in their experiments. Such differences in results are likely due to the ability of different strains of *D. melanogaster*, for example, wild‐type compared to laboratory strains, to cope with environmental stressors.

Fly responses to starvation were sexually dimorphic. Males exhibited higher resistance to starvation and thus survival when reared in a diet free of or with reduced bacterial load (the axenic, antibiotic treatments). Egg dechorionation had no effect on male resistance to starvation. In contrast, females exhibited increased resistance to starvation when their eggs were dechorionated; being reared in an axenic diet had no effect, and an antibiotic‐supplemented diet decreased female resistance to stress. From these results, it is clear that antibiotic has some deleterious effects on females when they are faced with starvation, and some beneficial effects on male resistance to starvation. Thus, there is a contradictory effect of antibiotic according to sex. Egg dechorionation and axenic rearing of the larvae increased resistance to starvation in females and males, respectively. However, depending on sex, removal of bacteria could be beneficial when starving. Different scenarios possibly explain this. Bacteria residing in the guts need to feed in order to develop, and may compete with the host for nutritional resources. An alternative explanation is that some bacteria may have deleterious effects on the host, and in their absence, the flies are healthier.

The presence/absence of bacteria in the diet during development of the fly also altered locomotion in relation to sex, while egg dechorionation had no impact. Females showed a decrease in their level of activity when reared in an axenic and/or antibiotic‐supplemented medium. This result demonstrates that bacterial feeding by females during development is essential for activity levels. Males are less affected by the absence of bacteria during development. Potentially females’ needs are higher than males due to egg production; bacteria may participate in this process either through the digestion of nutrients, or through the hormonal pathway. Indeed, *Lactobacillus plantarum* is known to control hormonal growth signaling (Storelli et al., 2011). It could be that the symbiosis between the fly and their gut microbiota is tighter in females than males, rendering females more susceptible to the absence of bacteria during development.

In addition to determining deleterious effects of treatments on the overall health and physiology of the fly, a key part of this study was confirmation of the effectiveness of each treatment. Our results showed that flies reared on a streptomycin diet had their gut bacteria completely eliminated; no bacteria were present on the plates. This result remained fairly consistent for the egg dechorionation, streptomycin treatment, in which two of the replicates were devoid of bacteria. One of these replicates, however, did contain some bacteria, though at low titer, and is likely to have resulted from contamination from another part of the *Drosophila* during dissection. The treatments containing streptomycin did, however, still possess a substantial amount of bacteria when the whole fly was analyzed, although less than the normal flies. This is to be expected, as adding streptomycin to the dietary media was designed to specifically eliminate the gut microbiota, rather than the entire *Drosophila* microbiota. Both treatments reared on axenic media contained similar numbers of colonies to the normal flies. Across all treatments, we identified the bacteria present as *Lactobacillus brevis*, a bacterium that has been previously found to dominate in flies with reduced bacterial diversity, as a result of being reared on a sterile diet (Broderick, Buchon, & Lemaitre, 2014).

An essential aspect of behavioral experiments relies on the ability to easily manipulate individuals when conducting an experimental design. In *Drosophila*, and other insect research, aspirators are commonly used to move individuals between treatments, as it allows for individuals to be manipulated without the use of carbon dioxide anesthesia, which has been shown to negatively impact on mating behavior in some species (e.g. Verspoor, Heys, & Price, 2015). Producing axenic or egg‐dechorionated individuals inhibits this ability to aspirate flies directly, in order to prevent external bacteria being transmitted onto the fly or their immediate environment, which could potentially confound experimental results. Therefore, we propose that the purpose of the experiment be an integral factor when considering which gut microbiota elimination method to choose; based on our results, we would suggest that the addition of streptomycin to the dietary media is the most favorable for behavioral research.

*Drosophila melanogaster* is one of the most useful and powerful models to study host–microbiota interactions. The fly harbors differing levels of bacterial diversity depending on rearing condition (e.g. natural vs. laboratory), but overall this diversity is disproportionately lower than in mammals. Thus, the fly is a highly convenient model for evaluating interactions between bacteria, and between bacteria and the host, and how these interactions affect the host. To date, most studies of the interactions of *D. melanogaster* with its microbiota have focused on the molecular dialog between them (Lhocine et al., 2008; Storelli et al., 2011); Buchon, Broderick, Chakrabarti, & Lemaitre, 2009). Our study highlights the need to take into account not only the molecular dialog, but also the final phenotypic effects of the interaction between the host and its microbiota, in terms of host fitness traits, as these could have strong evolutionary implications for host populations. It also demonstrates that the addition of streptomycin to the larval growth media effectively eliminates the resident bacteria within the *D. melanogaster* gut while resulting in the fewest non‐specific, deleterious effects in our host organism. However, it is important to consider that microbiota even within the same species/strains can differ between laboratories, so evaluating individual methods is necessary for a robust experimental design. Of equal importance is the consideration of the type of experiment performed. Adding low‐dose streptomycin to the dietary media is the most reliable and practical method of eliminating the gut bacteria, while still allowing easily manipulation of the host for behavioral experiments, and without introducing external bacteria. This method has the potential for widespread use for elucidating the understanding of host–microbiota systems, not only in *Drosophila*, but across all other insect systems.

## CONFLICT OF INTEREST

The authors declare no competing financial interests. Data has been deposited in Dryad

## AUTHOR CONTRIBUTIONS

CH, AL, AD, and ZL designed the experiment, and wrote the first draft of the manuscript. CH, FB, and LW conducted the experiment. CH, FB, and AL analyzed the data. All read and edited drafts of the manuscript.
